# Preclinical assessment of a modified Occlutech left atrial appendage closure device in a porcine model

**DOI:** 10.1038/s41598-021-82359-1

**Published:** 2021-02-04

**Authors:** Markus Reinthaler, Johannes Grosshauser, Tanja Schmidt, Juliane Unger, Ross Morgan, Friederike Zimmermann, Johannes Hartung, Claudio Seppelt, Denitsa Meteva, Wolfram Haider, Ulf Landmesser, Carsten Skurk

**Affiliations:** 1 Department of Cardiology, Charité University Medicine, Campus Benjamin Franklin, Hindenburgdamm 30, 12203 Berlin, Germany; 2 DHZK, Partner Site Berlin, Potsdamer Str. 58, 10785 Berlin, Germany; 3 Institute of Biomaterial Science, Helmholtz-Zentrum Geesthacht, Kantstrasse 55, 14513 Teltow, Germany; 4 Animal Core Facility, Charité University Medicine Berlin, Campus Virchow, Augustenburger Platz 1, 13353 Berlin, Germany; 5 Occlutech GmbH, Winzerlaer Str. 2, 07745 Jena, Germany; 6 Institute for Animal Pathology, Schoenhauser Strasse 62, 13127 Berlin, Germany; 7 Berlin Institute of Health, Anna-Louisa-Karsch-Straße 2, 10178 Berlin, Germany

**Keywords:** Cardiology, Interventional cardiology

## Abstract

Left atrial appendage (LAA) closure is being developed as an alternative for stroke prevention in patients with atrial fibrillation that cannot tolerate long-term oral anticoagulation. To assess the feasibility, safety, and performance of a novel modified Occlutech LAA closure device in a preclinical porcine model, the modified Occlutech modified Occlutech Plus LAA closure device was implanted in 12 female pigs (25–39 kg body weight) under fluoroscopic and transesophageal echocardiography (TEE) guidance. Procedural and technical success, as well as safety of LAA closure, were evaluated peri-procedurally and after 4, 8, and 12 weeks. Moreover, after 4, 8 and, 12 weeks animals were sacrificed for pathological analysis (e.g., thrombus formation, device ingrowth, endothelialization, and inflammation). All LAA closure devices were successfully implanted. On follow-up, no serious adverse events such as device-associated thrombus or translocalization/embolization were observed. A clinically non-significant pericarditis was observed in 4 animals at the time of autopsy. Endothelialization of the device was visible after 4 weeks, advanced after 8 weeks and completed after 12 weeks. Immunohistochemistry showed low amounts of inflammatory infiltration on the edges of the device. The results of this study indicate that implantation of a modified Occlutech LAA closure device is feasible with rapid endothelialization and low inflammatory infiltration in a porcine model. Human data are needed to further characterize safety and efficacy.

## Introduction

Improving stroke prevention in patients with atrial fibrillation (AF) is of paramount clinical importance^1^. Non-Vitamin K antagonists (NOACs) have become the standard of care for stroke prevention in AF patients^2–4^. However, approximately 20–30% of AF patients discontinue oral NOAC therapy within 24 months^2–4^ for reasons such as major bleeding, renal insufficiency and a perceived high bleeding risk. Moreover, current systemic anticoagulation therapy is still accompanied by a risk of major bleedings occurring at a rate of 2–3% per year although important high-bleeding risk patient groups were excluded from the phase III NOAC trials^2–4^. Accordingly, approximately 20% of patients with AF in need for stroke prevention therapy remain without oral anticoagulation in contemporary cohort studies^5–7^. The exclusion of the LAA from systemic circulation, a location prone to thrombus formation in AF patients^8^, may serve as an alternative therapy for stroke prevention in patients with AF who are not eligible for long-term oral anticoagulation therapy^9–12^.

Efficacy and safety of LAA occlusion (LAAO) have been analyzed in two randomized studies (anticoagulant eligible patients)^9,10^ and large-scale prospective observational registries (anticoagulation non-eligible patients)^13,14^. Five-year outcome data were combined in a meta-analysis^11^. LAAO was non-inferior to warfarin for the composite of stroke, systemic embolism, and cardiovascular/unexplained death. Moreover, differences in mortality, hemorrhagic stroke and major bleeding favored LAAO. In contemporary registries of LAAO the peri-procedural complication rate is approximately three percent^13,14^. Because of these data, numbers of LAA closure have considerably increased within the last years. Given the increase in AF patients in the future, the importance of LAAO as stroke preventive therapy might gain even larger importance.

Occlutech has developed an improved version of its LAA occluder. It consists of a self-expanding PU mesh covered conical lobe that obstructs the neck of the LAA and seals the ostium by its larger proximal diameter^15^, thereby preventing blood flow into the body of the LAA. The basic function of the device has already been investigated in a preclinical study^16^ as well as in a clinical trial^15^. An insufficient anchoring of the device was identified as a safety concern. Therefore, additional anchors were added to the design that support distal loops to ensure a safe fixation in the left atrial appendage. Moreover, the polymer surface was modified in order to improve device ingrowth and to accommodate the addition of the anchors.

The objective of the current study is to further evaluate the safety and efficacy as well as the biocompatibility of the Occlutech LAA occluder and to determine its performance.

## Methods

The study was performed at the animal core facility of Charité, CVK, in Berlin in accordance with the regulations of the national and international Animal Welfare Act and in accordance with local guidelines and laws and the ARRIVE-Guidelines. All experimental protocols were approved by the ethics committee of the Landesamt für Gesundheit und Soziales Berlin (LAGeSo, A 0185/18). The primary objective of this study was to verify basic functions of the Occlutech Plus LAA closure device in a porcine model:

- Procedural success: possibility of placement and deployment
- Technical success: sealing properties
- Safety: adverse events during and after implantation (incl. dislocation)
- Pathology: thrombus formation (chronic) and ingrowth of device

Primary endpoints were defined as:

1. The device is successfully positioned and anchored within the LAA sealing the orifice (leak < 5 mm).
2. Implantation is achieved without serious complications such as, thrombus, cerebral/systemic embolism or dislocation/embolization of implant.

Secondary endpoints were defined as:

1. Successful LAA closure (leak maximal 5 mm) at follow up.
2. Ingrowth of the implants in surrounding tissue.

### Occlutech LAA Plus left atrial appendage closure device

**Figure 1:**
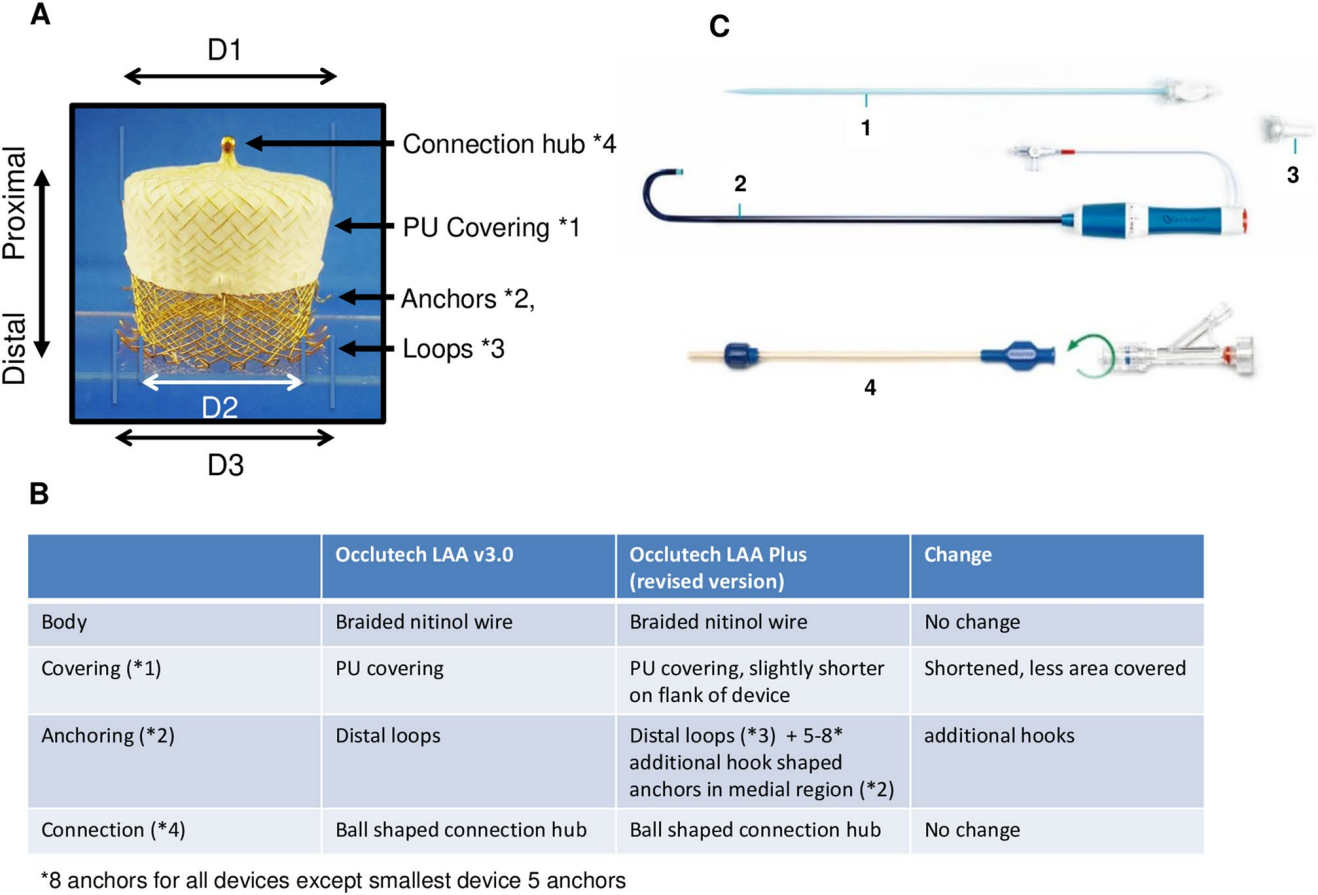
Occlutech Plus LAA closure device and delivery system. (A) The modified Occlutech LAA closure device consists of a proximal (PU membrane covered) and a distal (nitinol mesh) part. Distal positioned loops and newly developed anchors situated between the proximal and distal device parts secure anchoring within the LAA. The nitinol mesh has self-expanding properties ensuring self-modeling of the device within the LAA. The polyurethane (PU) covering serves as barrier excluding blood flow from the LA to the LAA. The connection hub serves as docking for the delivery system. B) Comparison of new device characteristics compared to the predecessor. C) Delivery system: the dilator (1) made from smooth conformable low friction radio-opaque material is inserted into the steerable delivery sheet (2) consisting of a thin braided wall and a soft flexible tip allowing bidirectional deflection up to 180°. The sheath has a hemostatic valve and can be connected by an adapter to the loader (4) made of transparent low friction material to allow inspection for air bubbles after pulling the occluder into the loader. Permission for usage of the image granted by OCCLUTECH Holding AG.

The modified Occlutech left atrial appendage closure device (Occlutech, Jena, Germany) consists of a conical-shaped, self-expandable Nitinol wire mesh that adapts to the shape of the LAA (Fig. 1). Distally attached loops and novel added additional anchoring elements on the device flank aid to keep the implanted device into position. The occluders outer surface is partially covered by a modified, non-woven, biostable Poly-Urethane (PU) layer.

### Study protocol

**Figure 2:**
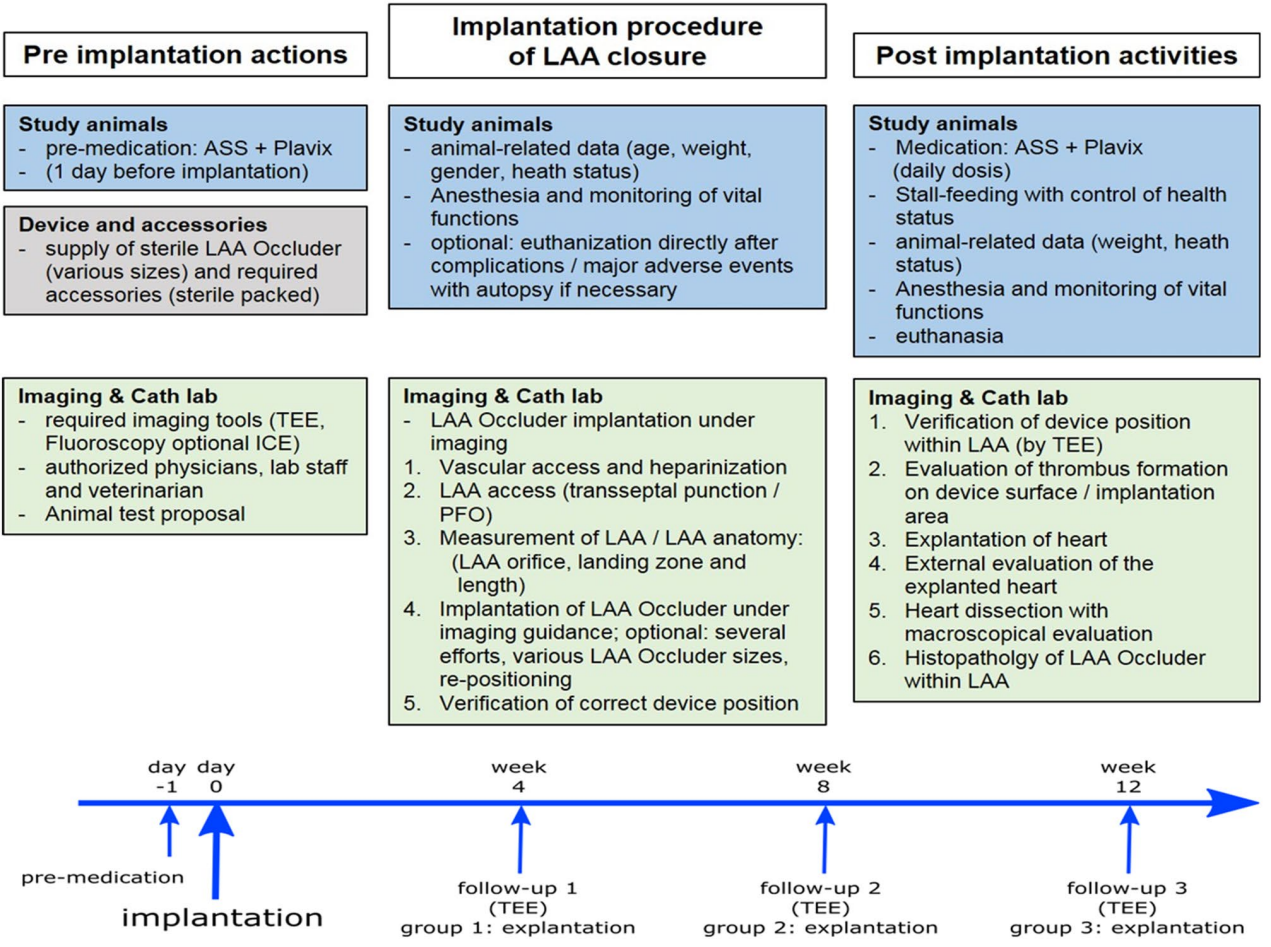
Study design. Timeline of pre-implantation actions, LAA occlusion procedure and post-implantation activities within the study protocol. 16 animals were used in the study, four were lost at day 0 (implantation). Animal groups 1, 2 and 3 consisted of four animals each that completed follow-up and histopathological evaluation.

A total of 16 healthy domestic female pigs (race: *Sus scrofa domesticus*) at an age of 2–4 months and a mean body weight of 33.3 kg were included. Because of an underrepresentation of challenging LAA shapes such as cauliflower and chicken wing in the canine model, pigs were used in our study to investigate save anchoring of the device. In Fig. 2 an overview of the design of the study is given showing pre- and post-implantation activities as well as planned procedural steps for implantation of the Occlutech LAA occluder.

### Preparation of animals

The day before the procedure animals received a 300 mg loading dose of clopidogrel as well as 100 mg of aspirin (Fig. 2). The animals received ketaminhydrochloride (10 mg/kg), xylazin (0.2 mg/kg), azaperon (120 mg/animal) and 0.02 mg atropine i.m. Analgosedation was carried out by midazolam (1 mg/kg/h) and fentanyl (12–13 µg/kg/h). An additional bolus of propofol (20 mg) was given for intubation. Animals were ventilated on a volume-regulated respirator, etCO2 was maintained at physiological levels, arterial pressure was monitored by an arterial line (common femoral artery) and urine output was measured. For peri-interventional antibiotic prophylaxis the animals received Sultamicillin 3 g i.v. Additionally, animals received 150 mg amiodarone, 100 mg lidocaine and 1 g magnesium i.v.as loading dose and amiodarone was continued per infusionem during the procedure.

### Implantation technique

**Figure 3:**
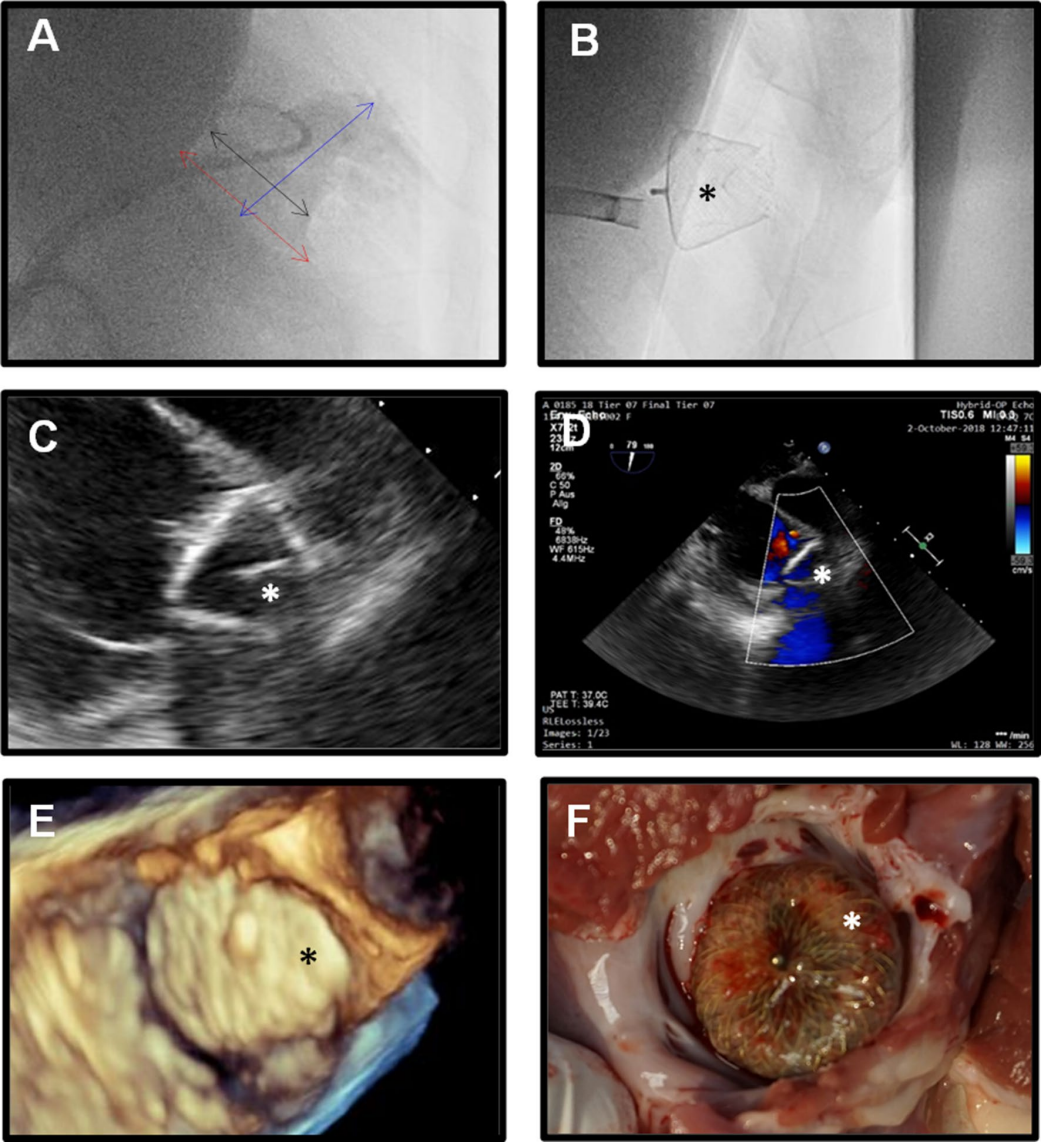
Imaging and morphological examination at follow up. Fluoroscopic images illustrating LAA anatomy and measurements for device implantation (orifice- red arrow, landing zone- black arrow, LAA length- blue arrow) **(A)** as well as device implants (asterisk) **(B)**. Anchoring of the device and sealing of the LAA was confirmed by (Doppler)- TEE imaging **(C,D)**. 3-D TEE imaging illustrates occluder position (asterisk) inside the LAA ostium **(E)**. Macroscopic evaluation of LAA device implants (asterisk) was performed **(F)**.

The common femoral vein puncture was guided by ultrasound. A vascular closure system (Perclose) was pre-installed. The left atrium was accessed via standard trans-septal puncture using a Brockenbrough needle (St. Jude BRK) under fluoroscopic and TEE guidance or via an existing PFO. A heparin bolus (5000 IE) was intravenously administered when the trans-septal sheaths were introduced into the right heart followed by a continuous infusion to keep the activated clotting time during the procedure > 250 s. Fluoroscopy imaging of the LAA was obtained by contrast injection via a pigtail catheter. The LAA orifice diameter, landing zone and length was measured using TEE and contrast fluoroscopy (Fig. 3). A device size with a D1 (diameter of proximal part) approximately 1–4 mm larger than the LAA landing zone was chosen. All devices were implanted with the Occlutech steerable guiding sheath (OSGS). Due to its flexibility and the option to alter the angulation of the distal catheter tip, this device allowed successful delivery of the occluders in all animals, irrespective of the pre-existing anatomical conditions.

In brief, the sheet was placed in the left superior pulmonary vein via an Amplatzer Super Stiff wire. After bringing the steerable guiding sheath into its position the tip of the sheath was carefully advanced into the LAA until it reached the landing zone where the device was deployed. Correct placement of the device and occlusion was confirmed by tug test, TEE and contrast fluoroscopy. Detailed information about implant location, protrusion, leakage or gaps, potential impacts on surrounding anatomical structures and observed adverse events during the interventional procedure was obtained. In case of incomplete anchoring, leakage or protrusion a repositioning of the occluder was attempted until the best possible result was observed, or the device was recaptured and replaced with a different size. Following implantation of the device, the delivery system and pusher were retrieved, the femoral vein was ligated with a Perclose closure system and the skin was closed in two layers. Post-procedural medication consisted of aspirin 100 mg and clopidogrel 75 mg per day.

### Follow-up

Animals were assigned to one of three follow-up groups (i.e., 4, 8 and 12 weeks, Fig. 2). On follow-up, animals underwent a general health assessment (veterinary), were then anaesthetized (midazolam/fentanyl) and the device position, any potential leakage or thrombus formation on the device was visualized by TEE. After the last follow-up examination, the animals were euthanized using potassium chloride. The hearts were explanted and a visual examination of the outside of the heart and the situs of the occluder were carried out. Moreover, thrombus formation and biocompatibility and ingrowth of the device were macroscopically characterized (Fig. 3).

### Histopathological examination

Devices were explanted and fixated in 5% formalin. For further histo-pathological analysis (i.e., evaluation of device endothelialisation and peri-device inflammation) 3 samples were taken:

- Sample 1: Tissue from inside of the occluder
- Sample 2: Tissue from the occluder’s cover
- Sample 3: cardiac tissue around the anchors.

Tissue was embedded in paraffin and cut on a rotation microtome in 2 μm tissue slices.

Tissue samples of the myocardium in the area of device anchoring were embedded in Technovit 9100 and cut with a thickness between 35 and 40 μm. All samples were stained by hematoxylin–eosin and visualized on an Olympus BX 51 microscope at × 20, × 50, × 100, × 200 and × 400 magnification. Representative slides were chosen for the manuscript observed in every animal investigated.

### Implantation success

Acceptance criteria for a successful implantation of the Occlutech LAA Plus occluder were defined as procedural success determined as successful deployment of LAA occluder without the occurrence of major complications such as device embolization, pericardial effusion (using tamponade), significant device-related inflammation or irritation (evaluated macroscopically and histologically after explantation), death, bleeding and vascular complications. The technical success was characterized as acute sealing rate / LAA closure rate after follow-up periods of 4, 8 and 12 weeks defined as LAA closure without a leak > 5 mm.

## Results

### Implantation success, adverse events and complications during the implantation procedure

Out of 16 animals used for the study, one animal deceased due to pericardial tamponade after transseptal puncture because of difficulty to visualize the interatrial septum in a complex porcine anatomy. In the remaining 15 animals, successful implantation of an Occlutech Plus device was performed. One animal suffered from air embolism because of trapped air in the delivery sheet after successful device implantation. Two animals died from sudden cardiac death while awakening from anaesthesia. Autopsy of these animals did not reveal remarkable pathological findings (no device perforation, no device embolization, and no pericardial effusion); physical stress regarding withdrawal of anesthesia is therefore the most likely reason for death in these animals. All other animals survived the procedure and were well at follow-up. No direct LAA device related adverse events (e.g. unintended LAA device disconnection, device embolization or LAA tissue damage due to designed anchors/loops of the Occlutech LAA Plus Occluder) were observed during device handling, repositioning or device replacement within the LAA.

All implantation procedures were performed without LAA device embolization, atrial clotting or thrombus formation on the occluder surface. Moreover, no apparent strokes or bleedings were observed. Details of the implanted devices are shown in Table 1. According to the measurements and the devices employed an average oversizing of 3–4 mm was used. This is at the high end of the initially recommended 1–4 mm. However, complete sealing was achieved in most cases (Table 2). Oversizing might explain the not clinically significant discrete protrusion of devices into the LAA in 33% of cases.

**Table 1:** LAA anatomy, sizing and implants used in the study.

Follow-up	Study no	LAA			Oversizing		LAA anatomy	LAA occluder size
		Orifice [mm]	Landing zone [mm]	Length [mm]	Orifice [mm]	Landing zone [mm]		
4 week group	1	18	14 (TEE) 18 (Fluor)	26	6	6	Chicken wing	LAA 24 (PD2018-052–04)
	2	17 (Fluor)	17	21	5	7	Chicken wing Short landing zone	LAA24 (PD2018-052–20)
	3	18 (Fluor)	17	13	5	7	Chicken wing	LAA24 (PD2018-052–01)
	4	21 (TEE)	15	13	0	6	Chicken wing	LAA21 (PD2018-051–12)
8 week group	5	16	18	10	5	3	One lobe, difficult to distinguish LA and LAA	LAA21 (PD2018-051–03)
	6	14–18	18	21	6	6	One lobe Chicken wing	LAA24 (PD2018-052–06)
	7	20 (Fluor)	18 (TEE) 19 (Fluor)	19	1	3	Chicken wing	LAA 21 (PD2018-051–20)
	8	18	13 (TEE) 16 (Fluor)	21 (Fluor)	0	2	Two lobes Cactus	LAA 18 (PD2018-050–18)
12 week group	9	18	15	24	0	3	Large central lobe with short neck, at least two further small lobes	LAA18 (PD2018-050–02)
	10	19 (TEE)	16 (Fluor)	24	2	5	Cauliflower	LAA21 (PD2018-051–09)
	11	17 (TEE)	16 (TEE) 19 (Fluor)	N/A	2	3	Chicken wing with multiple lobes	LAA 21 (PD2018-051–19)
	12	16	16	20	3	2	One lobe	LAA 18 (PD2018-050–21)

**Table 2:** Technical properties of LAA closure during follow-up.

Follow-up	Study no	Adaptation to LAA		Sealing rate		Device compression		Device protruding in to LA		Atrial clotting	
		Implant	FU	Implant	FU	Implant	FU	Implant	FU	Implant	FU
4 weeks	1	Good	Good	No leak (0)	No leak (0)	No	No	Yes (~ 2 mm)	Yes (~ 2 mm)	No	No
	2	Moderate	Moderate	No leak (0)	No leak (0)	Distal	Distal	No	No	NO	No
	3	Good	Good	No leak (0)	No leak (0)	Distal	Distal	No	No	No	No
	4	Moderate	Good	No leak (0)	No leak (0)	Distal	Distal	Yes (~ 3 mm)	Yes (~ 3 mm)	No	No
8 weeks	5	Good	Good	No leak (0)	No leak (0)	No	No	No	No	No	No
	6	good	good	no leak (0)	small leak (2)	distal (approx. 50%)	distal (approx. 50%)	yes (~ 2 mm)	yes (~ 2 mm)	no	no
	7	Good	Good	Trivial leak (1)	Small leak (2)	No	No	Yes (~ 2 mm)	Yes (~ 2 mm)	No	No
	8	Good	Good	No leak (0)	N/A	No	No	No	No	No	No
12 weeks	9	Good	Good	No leak (0)	Small leak (2)	Distal	Distal	Yes (~ 4 mm)	Yes (~ 4 mm)	No	No
	10	Good	Good	No leak (0)	No leak (0)	Distal	Distal	Yes (~ 2 mm)	Yes (~ 2 mm)	No	No
	11	Good	Good	Trivial leak (1)	No leak (0)	Distal	Distal	Yes (~ 2 mm)	Yes (~ 2 mm)	No	No
	12	Moderate	Good	Small leak (2)	Small leak (2)	Distal	Distal	Yes (~ 2 mm)	Yes (~ 2 mm)	No	No

### Follow-up examinations of the animals

After a period of 4, 8 and 12 weeks a follow-up examination was performed (Fig. 2). These examinations consisted of a general health assessment of the animals and a TEE evaluation of the device. By TEE imaging the device position, any potential leakage or thrombus formation on the device and the presence of pericardial effusions was assessed. During follow-up, no adverse events other than those already reported at time of implantation (i.e., new AEs) were recorded. The specifics for each time point are listed in Table 2. Of note, no device associated thrombus, no device dislocation/embolization, no device failure and no clinically relevant pericardial effusions were detected.

The sealing of the LAA was defined by the size of any remaining leaks, rated from 0 (no leak), 1 (trivial leak < 3 mm), 2 (moderate leak 3–5 mm), and 3 (large leak > 5 mm). The acceptance criteria were defined as any leak except for large leaks. After the follow-up period 8 (67%) of the implants showed no leaks (grade 0), while in 4 animals (33%) trivial leaks (grade 1) were observed. Taken together, no new device leaks were documented by TEE Doppler during follow-up. Complete sealing of the different and sometimes challenging LAA anatomies was achieved in 100% of the cases. Protrusion (i.e., device shoulder > 2 mm after implantation) was detected in 33% of cases. However, only one case of protrusion > 3 mm was visualized and the position of the device did not change during follow up. These data indicate safe and secure anchoring of the modified Occlutech closure device within the LAA.

### Euthanasia and autopsy of the heart

**Figure 4:**
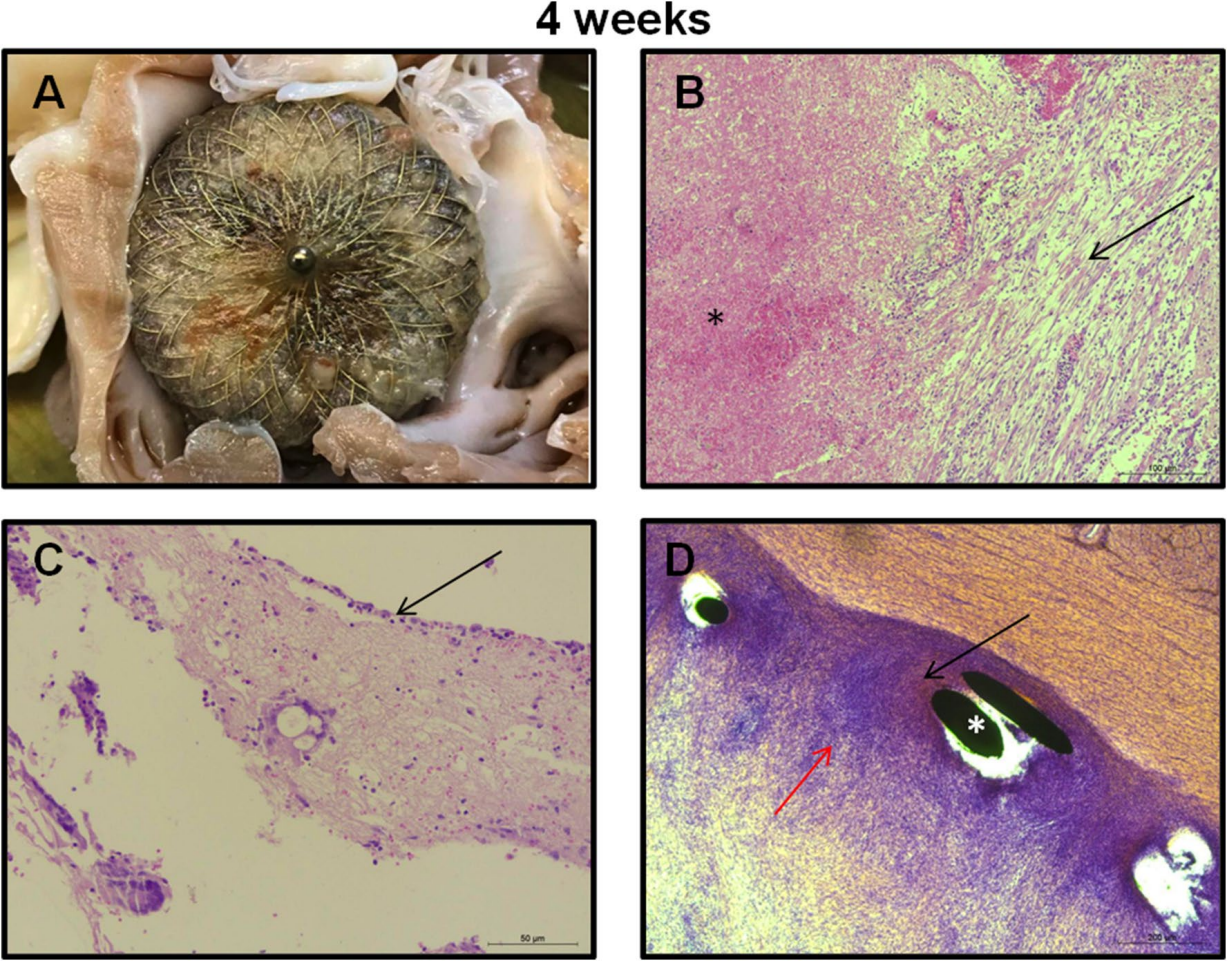
Histopathological examination of device and surrounding tissue at follow up. *4 wk fu: (A)* *Macroscopic evaluation* Device in situ. Complete sealing within the LAA ostium. Fibrin accumulation on the surface of the device. **(B)** *Thrombus* Thrombus in the border areas consists of loose connective tissue with erythrocytes (arrow) as well as moderate numbers of inflammatory cells (PMN, lymphocytes, few multinucleated giant cells). Most of the thrombotic material consists of coagulated blood (asterisk). **(C)** *Membrane* The cover of the PU membrane shows discrete connective tissue proliferation. In an islet-like pattern the connective tissue is thickened (infiltration with PMN and lymphocytes). On the connective tissue surface endothelial cells can be identified (arrow). **(D)** *Anchoring* Ingrowth of the device (struts indicated by asterisk) can be characterized by medium strength connective tissue (red arrow). A discrete to moderate inflammatory reaction consisting of PMN and lymphocytes can be depicted (black arrow). B-D hematoxylin–eosin (HE) staining.

**Figure 5:**
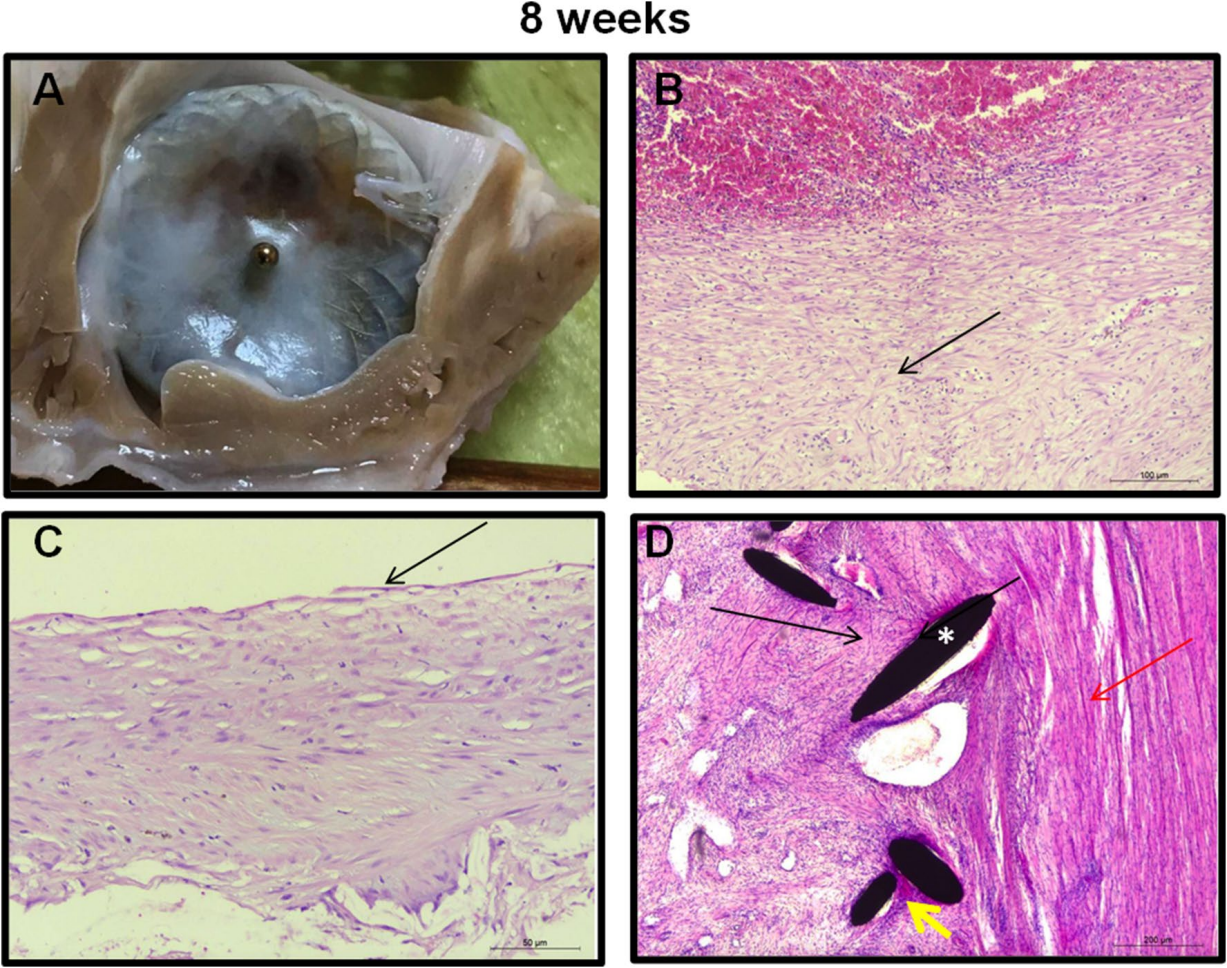
Histopathological examination of device and surrounding tissue at follow up. 8 wk fu: **(A)** *Macroscopic evaluation* Device in situ. Complete sealing within the LAA ostium. Connective tissue progresses on the device surface. **(B)** *Thrombus* Thrombus consists of loose connective tissue with nest-like calcifications (arrow). Plaques of lymphocytes and diffuse macrophages containing hemosiderin can be appreciated. Connective tissue shows collection of erythrocytes with good vascularization. **(C)** *Membrane*: The membrane is covered with connective tissue that is covered by an endothelial monolayer (arrow). **(D)** *Anchoring* At the ingrowth area (struts indicated by asterisk) vascularized connective tissue (black arrow) with a discrete lymphocytic inflammation (yellow arrow) is determined. The surrounding myocardium (red arrow) does not show fibrosis. B-D hematoxylin–eosin (HE) staining.

**Figure 6:**
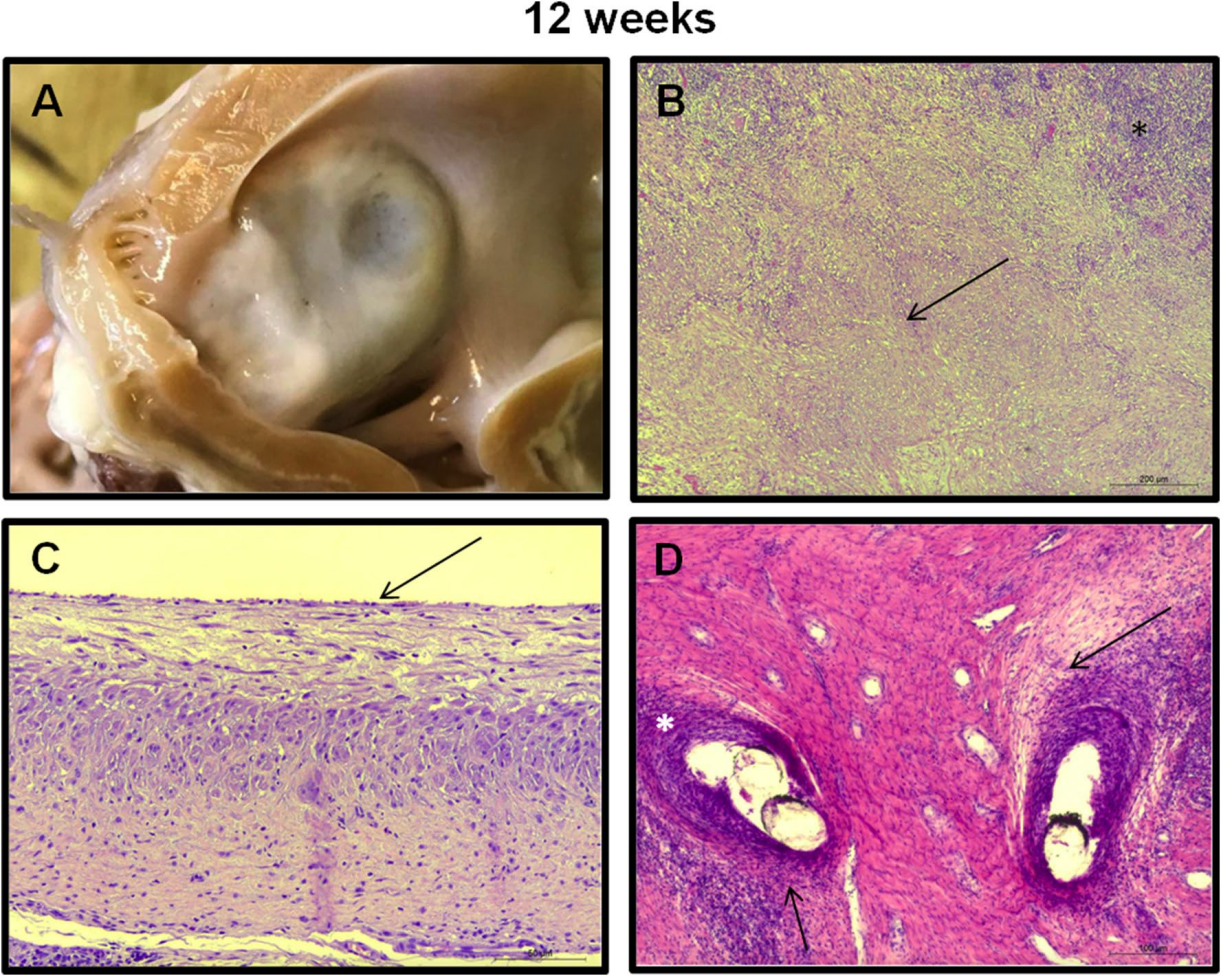
Histopathological examination of device and surrounding tissue at follow up. 12 wk fu: **(A)** *Macroscopic evaluation* Device sealing the LAA ostium is depicted. Connective tissue covered by an endothelial layer completely fills the device surface. **(B)** *Thrombus* Thrombus completely organized by connective tissue (arrow). **(C)** *Membrane* The membrane is completely covered with connective tissue that is covered by an endothelial monolayer (arrow). **(D)** *Anchoring I*n the areas of device ingrowth (black arrow) a cell-rich connective tissue developed with discrete diffuse inflammation consisting of PMN and lymphocytes (asterisk). B-D hematoxylin–eosin (HE) staining.

After the last follow-up examination, animals were euthanized (Fig. 2). The hearts were then explanted and a visual examination of the outside of the heart and the situs of the occluder following dissection was performed. Special care was taken for any signs of cardiac injury (i.e., perforation), inflammation (i.e., diffuse or localized pericarditis), pericardial effusion, device perforation/dislocation and thrombus formation, as well as the rate of tissue coverage on the device surface. Macroscopically, no apparent inflammation was visible on the surrounding endocardium. No thrombus was detected on the luminal surface of any device (Table 3). All devices were securely embedded in the LAA. Complete sealing of the LAA by the device was confirmed in all animals. Some device protrusion was detected, however, even in these cases the PU layer of the device was covered with a white tissue layer. Polyurethane is known for its good biocompatibility. It has improved processing capabilites which make it possible to directly attach the fibres to the braided device resulting in a non-woven porous fabric which facilitates instant sealing and good ingrowth of the LAA. The non-PU covered lower parts of the device were always securely embedded in the LAA surrounding tissue (Suppl. Fig. S1A, arrow). After 4 weeks significant portions of the proximal occluder surface were covered with tissue (Fig. 4). After 8 weeks, the tissue covering reached more than 50% of the surface in 3 out of the 4 animals (Table 3, Fig. 5). After 12 weeks all or the majority of the device surfaces were covered with a layer of tissue in all experimental animals (Table 3, Fig. 6). In none of the cases damage to the proximal polymer cover was observed. However, owing to the thick layer of tissue, not in all cases the covering was visible. The connector pin has been identified a critical structure for thrombus formation. In our study, the pin was covered by neoendothelium in one animal 8 weeks following LAAC, while 3 animals showed coverage after 12 weeks. Retention hooks were seen embedded into the appendage walls. In some animals, the distal loops of the devices penetrated the LAA wall with epicardial fibrous healing and without clinical relevant hemorrhagic pericardial effusions at any time at follow-up (Suppl. Fig. S1C). Of note, pericarditis was seen on macroscopic examination in 4 animals. However, these findings did not result in adverse clinical events (Table 3).

**Table 3:** Histopathologic examination of study groups.

Follow- up	Study no	External heart evaluation					
		Perforation /pericarditis	Signs of inflammation	Thrombus formation	Infection/Irritation	Endothelial growth	PU covering integrity
4 weeks	1	no	no	no	no	0–10%	fully covered
	2	No	No	No	No	10–50%	fully covered
	3	Diffuse pericarditis (ventricle)	No	No	No	~ 10% of LAA device surface	Fully covered
	4	No	No	No	No	Up to 10% of LAA-device surface	Fully covered
8 weeks	5	Pericardial thickening	Yes	No	Infection: mild signs	50%—full coverage of LAA device surface	Fully covered
	6	No	No	No	No	10—50% of LAA device surface	Fully covered
	7	No	No	No	No	50%—full coverage of LAA device surface	Not visible
	8	No	No	No	No	50%—full coverage of LAA device surface	not visible
12 weeks	9	Localized pericarditis, pericardial adhesion	No	No	No	50%—full coverage of LAA device surface	Fully covered
	10	Diffuse pericarditis	No	No	No	50%—full coverage of LAA device surface	Fully covered
	11	Multiple pericardial adhesions	No	No	No	50%—full coverage of LAA device surface	Fully covered
	12	Diffuse pericarditis, mainly ventricular	No	No	No	50%—full coverage of LAA device surface	Not visible

The LAA cavity distal to the cover was filled up with organized thrombus. The organization of this thrombus progressed over time, at 12 weeks follow-up all thrombotic material was resorbed and displaced by connective tissue (Suppl. Fig. S2). The claws of the umbrella were well opposed to the native appendage walls and were covered again by neointima, with no evidence of tissue necrosis (Suppl. Fig. S1B). Taken together, the results of the pathologic-anatomical examination showed a rapid ingrowth of the device without tissue injury.

### Histopathological examination

The aim of the histopathological study was to assess device ingrowth and the biocompatibility by microscopic examination. All devices were partially or fully overgrown with tissue dependent on time of follow-up (i.e., 4 vs. 12 weeks). This connective tissue grows from the periphery to the center of the occluder in a time dependent manner (Figs. 4, 5, 6). In the center, this tissue is often thin layered consisting of immune cells. Especially in the beginning of the ingrowth process, multi-nucleated giant cells were determined in higher numbers. Of note, at 4 weeks after implantation low numbers of connective tissue cells and parts of an endothelial layer appear on the luminal PU surface. On histological analysis the early inflammatory cell adhesion is substituted by thin layers of connective tissue cells that will eventually be covered by an endothelial cell layer. Already 8 weeks post-implantation, this layering is complete (Fig. 5).

At the time of device explantation in the 4-week follow-up animals two of the devices showed limited covering of less than 10% with endothelial cells indicating ingrowth, while the other two already exhibited an endothelial cover of up to 50% of the surface area (Table 3).

At the time of device explantation of the 8-week-follow-up animals one device was fully covered, while three were only partially overgrown. Two of the partially covered devices exhibited an almost complete endothelial monolayer and only a small portion around the connection ball was still visible. One device was covered in the area close to the LAA orifice (Table 3). At the time of device explantation of the 12-week follow-up animals three devices were completely covered with a tissue layer, while one presenting with a large shoulder was only partially covered with endothelial tissue. From these observations it can be assumed that endothelial tissue starts to cover the device at points of contact with the LAA tissue and progresses from there towards the center of the device over time.

The PU membrane that is sealing the left atrial appendage ostium cannot be visualized histologically. In the distal part of the occluder where the anchors and loops anchor the device in the LAA slow proliferating connective tissue was detected. This tissue is well confined to the device and delineated from myocardial tissue. The growth of connective tissue is directed inwards to the device volume, where thrombotic material is organized (Suppl. Fig. S2). Connective tissue inside the occluder can be visualized as soon as 4 weeks after implantation and its formation does not appear to change much over time (i.e., 12 weeks of follow-up). Since the growth of connective tissue is directed inwards, effects on surrounding tissue after implantation seem to be neglectable. Apart from the connective tissue the adjacent surroundings of the nitinol struts were surrounded by a discrete to moderate inflammatory infiltration. Appearance of follicle-like lymphocytic structures indicated chronic inflammation that was determined 4 weeks, 8 weeks and 12 weeks after implantation (Figs. 4, 5, 6). The composition of this infiltration did not change over time. Considering the small degree and extent of this inflammation clinical symptoms are not expected. Taken together, the results of the histopathological examination indicate a good biocompatibility of the device at 4–12 weeks of follow-up.

## Discussion

In this preclinical porcine model, the basic functions of a modified Occlutech Plus LAA occluder were studied. In summary, a safe and successful implantation of the LAA closure device was achieved. In all study animals the pre- defined acceptance criteria were met. All adverse events observed during implantation were attributed to the complex anatomy of the animals or problems handling the accessories. One animal died because of air embolism since air was trapped in the delivery sheet. One animal had to be euthanized because of pericardial effusion after transseptal puncture. Two animals deceased after successful implantation of the device with complete sealing of the LAA while awaking from anesthesia without remarkable findings on autopsy. Scheduled transesophageal echocardiographic examinations at follow-up revealed no significant pericardial effusion, device associated mitral valve dysfunction or pulmonary venous obstruction. Gross pathological examinations showed that all devices were appropriately placed within the LAA, with complete sealing of the LAA ostium.

Percutaneous left atrial appendage closure is used as alternative for stroke prevention in AF patients that are ineligible for long-term oral anticoagulation^1^. Presently, many randomized controlled trials are recruiting patients potentially expanding the indications and increasing numbers for LAAC^12^ in the future. During the last five years, new CE marked devices were introduced to the market in order to improve safety and efficacy of the procedure (i.e., Lambre, Ultraseal, WATCHMAN FLX)^12^. In prospective registries, > 95% of patients could be successfully treated with an Amplatzer Amulet or WATCHMAN device, presently the most common used LAA occluders^13,14^. The peri-procedural complication rate currently constitutes around 3%^12^. However, both occluders still have their limitations. The WATCHMAN device is not suitable for patients with shallow LAA anatomy due to the length of the device. The repositioning flexibility of the device is limited since recapturing the device beyond the level of retention barbs is not recommended because of the risk of damaging the barbs. On the other hand, there is a tendency for the lobe of the Amplatzer Cardiac Plug to jump forward to proximal LAA after deployment and thereby demanding extra skills from the implanting physicians^17^. Moreover, both devices have to be delivered via non-steerable, relatively large-sized sheaths, which are inherently associated with more vascular and embolic complications^18,19^. The Occlutech Plus LAA closure device consists of a self-expanding, self-modeling flexible soft nitinol body with low radial force that results in better modeling and adaptation to the LAA wall resulting in complete LAA closure. Of note, the structure of the device allows implantation in the so-called fish-ball technique, increasing the safety of the procedure^20^. In the present study three attempts or more were needed to bring the device in its final position due to the complex anatomy in some of the animals. However, in all cases the device was implanted successfully completely sealing the LAA. No damage to the polymer covering or the device sealing properties were observed. Therefore, retracting the device into the sheath for repositioning is safe. The assembly device, pusher, loader and delivery sheath could be handled well in order to access the LA, deliver the device to the LAA and deploy or retract the device. Therefore, the device is considered safe and usable. In particular, in cases where the LAA was difficult to reach the steerable guiding sheath was of great advantage due to its flexibility to rotate 180° to reach the LAA thereby facilitating the procedure. The sheet is also usable for transseptal puncture. Therefore, a change to a delivery sheet is not necessary potentially leading to shorter procedure times and prevention of complications.

On follow-up in particular, no occluder embolization or device failure were observed. Neither by TEE nor by macroscopic evaluation of the explanted heart was any thrombus on the device determined. Veterinary exams showed no other clinically apparent adverse events. Due to the design of the device and the oversizing, more or less protrusion into the LA lumen occurred. However, all devices showed a time dependent neo-intima formation that was fully endothelialized after 12 weeks. The lower, not PU covered waist region provided a secure anchoring indicated by the strength needed to separate this part of the occluder from surrounding tissue at pathological examination and collagen embedment on histological investigation. In three pigs the distal loops used for anchoring penetrated the distal LAA lobe. These loops are designed in an atraumatic manner to engage with trabeculae to anchor the distal part of the device within the LAA. Importantly, no bleedings, significant pericardial effusions or other damage to the LAA was visible and the animals were clinically unremarkable with normal weight gain. These observations are in line with earlier studies^16^ that described penetration of some distal loops without serious complications such as pulmonary artery perforation, pericardial effusion or pericarditis^21^. They were therefore considered clinically insignificant.

The device healing process appeared to be safe in that no thrombus formation of any sort (loose or attached) was found on the PU membrane at the observed follow-up time points by TEE or at macroscopic evaluation and the end of the study. Fibrin on the membrane was thin and laminar, covering the whole surface and becoming a matrix for inflammatory cells and fibroblasts after implantation and endothelial cells during the 12-week follow-up. No clinically significant inflammatory reaction and no necrosis in adjacent cardiac tissue following implantation of the device were observed. In all analyzed cases was tissue continuity seen at the device edges, hence leaving no possibility for any contents of the appendage body to exit the cavity of the appendage. Furthermore, adequate device integration into the wall was observed, therefore suggesting a very low risk of embolization in the observed cases. Aforementioned healing events initiate with a fibrin-thrombus deposition, where initially an unorganized structure can be observed, which in the course of time develops into a dense and organized structure. Subsequently controlled inflammation, consisting of macrophages and lymphocytes, occurs which results in resorption of the thrombus. The generation of fibrous tissue, along with the infiltration of smooth muscle cells lead to the endothelial coverage that ultimately resembles the endocardium, hence resulting in a final, biocompatible blood-contacting interface.

Taken together, our data show a rapid ingrowth and healing process following Occlutech LAA occluder implantation. Efficacy and safety were comparable or superior to preclinical models with other LAA devices^16,22–24^.

### Limitations of the study

This experimental animal study had a small sample size number and short follow-up period. However, the predecessor device was investigated in preclinical and clinical studies with regard to biocompatibility, usability and safety. Structural changes included additional hooks and a modified PU membrane. Hence, it was deemed acceptable to use a low sample number. Notably, the anatomy and structure of the porcine LAA is different from the human LAA that should be considered when results of the study are applied to clinical practice. While the data of this proof-of-concept study are encouraging with regard to safety and performance, further investigations should address different anti-thrombotic therapeutic schemes, shortening of post-implantation anti-thrombotic therapy and compare the device safety and performance to other available LAAC devices. Considering previous experience with the Occlutech device and our data it is recommended to investigate the safety and performance in a clinical trial.

## Conclusion

Taken together, our preliminary data suggest that the modified Occlutech LAA occluder is feasible and can be implanted with a high success rate in a porcine model.

## Supplementary Information

Supplementary Figure 1.

Supplementary Figure 2.

Supplementary Figure Legends.
