# Supplementary figures and images for: Preclinical assessment of a modified Occlutech left atrial appendage closure device in a porcine model
Source: Sci Rep. 2021 Feb 4;11:2988. doi: 10.1038/s41598-021-82359-1 (PMC7862256; doi:10.1038/s41598-021-82359-1)

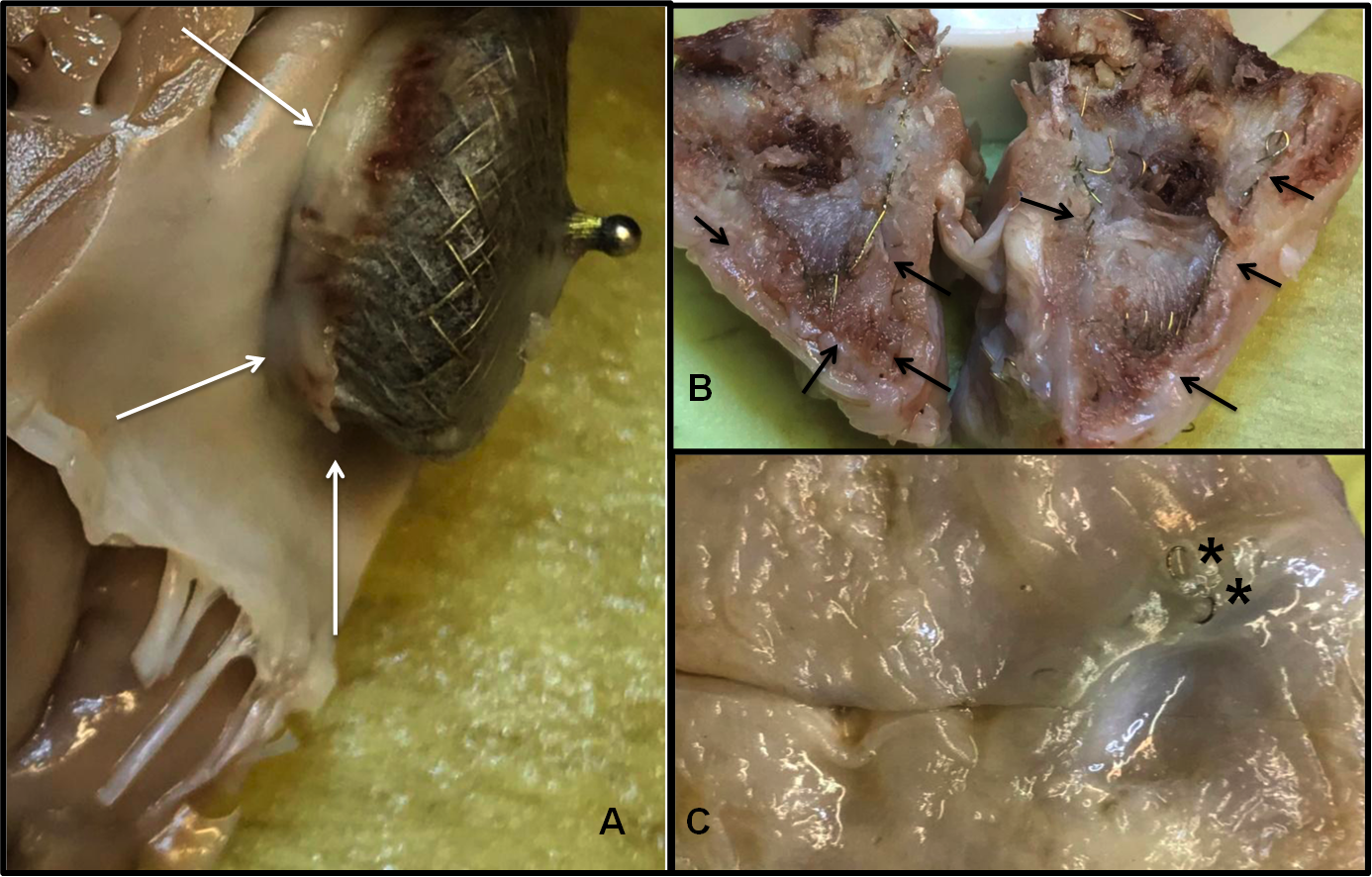

Supplement: Supplementary file 1 — Supplementary Figure 1. [file 41598_2021_82359_MOESM1_ESM.tif]

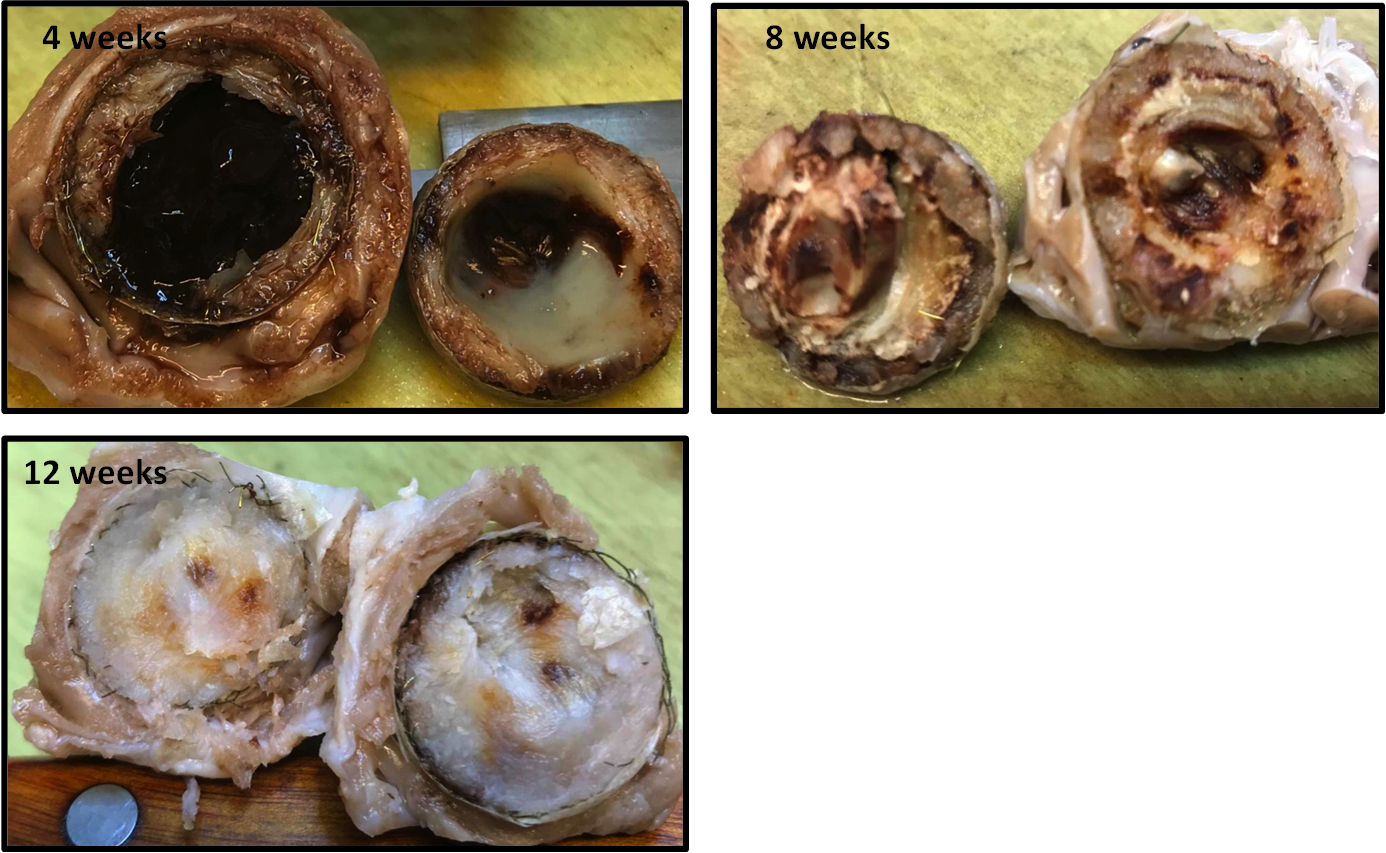

Supplement: Supplementary file 2 — Supplementary Figure 2. [file 41598_2021_82359_MOESM2_ESM.tif]
