# Supplementary material for: Preclinical assessment of a modified Occlutech left atrial appendage closure device in a porcine model
Source: Sci Rep. 2021 Feb 4;11:2988. doi: 10.1038/s41598-021-82359-1 (PMC7862256; doi:10.1038/s41598-021-82359-1)
Supplement: Supplementary file 3 — Supplementary Figure Legends. [file 41598_2021_82359_MOESM3_ESM.docx]

**Suppl. Figures:**

**Suppl. Figure 1: Macroscopic Evaluation following LAAO. *(A)*** Despite protrusion of the occluder into the LAA the device is covered by am PU membrane. Ingrowth at the site of anchoring is shown (white arrows). ***(B)*** LAA cut open: anchoring of the device (struts and hooks—black arrows) is supported by cardiac and connective tissue. ***(C)*** Penetration of distal hooks through the LAA wall (asterisk). No sign of pericarditis or bleeding was appreciated.

**Suppl. Figure 2: Phases of organization of LAA thrombus post- device implantation.** Initial coagula are organized by ingrowth of connective tissue. 12 weeks after implantation, the former thrombus completely consists of newly grown connective tissue.
